# A Novel CNN-based Bi-LSTM parallel model with attention mechanism for human activity recognition with noisy data

**DOI:** 10.1038/s41598-022-11880-8

**Published:** 2022-05-12

**Authors:** Xiaochun Yin, Zengguang Liu, Deyong Liu, Xiaojun Ren

**Affiliations:** 1 Shandong Provincial University Laboratory for Protected Horticulture, Weifang Key Laboratory of Blockchain on Agricultural Vegetables, Weifang University of Science and Technology, Weifang, 262700 China; 2 The College of Computer Science and Engineering, Shandong University of Science and Technology, Qingdao, 266590 China; 3 Shandong Software Engineering Technology Center, WeiFang University of Science and Technology, Shouguang, 262700 China

**Keywords:** Computer science, Information technology

## Abstract

Boosted by mobile communication technologies, Human Activity Recognition (HAR) based on smartphones has attracted more and more attentions of researchers. One of the main challenges is the classification time and accuracy in processing long-time dependent sequence samples with noisy or missed data. In this paper, a 1-D Convolution Neural Network (CNN)-based bi-directional Long Short-Term Memory (LSTM) parallel model with attention mechanism (ConvBLSTM-PMwA) is proposed. The original features of sensors are segmented into sub-segments by well-designed equal time step sliding window, and fed into 1-D CNN-based bi-directional LSTM parallel layer to accelerate feature extraction with noisy and missed data. The weights of extracted features are redistributed by attention mechanism and integrated into complete features. At last, the final classification results are obtained with the full connection layer. The performance is evaluated on public UCI and WISDM HAR datasets. The results show that the ConvBLSTM-PMwA model performs better than the existing CNN and RNN models in both classification accuracy (96.71%) and computational time complexity (1.1 times faster at least), even if facing HAR data with noise.

## Introduction

Human activity recognition^1^ is to classify and recognize the movement behaviors by analyzing the information of human activities, which has great commercial value and scientific research significance in the fields of human-computer interaction, covid-19 tracking and public safety. Nowadays, most smartphones are equipped with quite rich embedded sensors, such as acceleration sensor, gyroscope sensor, magnetic sensor, etc., which can provide enough training data of human activities with the advantages of portable, low power consumption and low cost. And more, they can provide enough computing units for deep learning models. Therefore, it is suitable that smartphones are chosen as the terminals for human behavior recognition. They can not only collect data conveniently, but also preprocess data, extract features and recognize real-time behavior. Gradually, human behavior recognition based on smart phone sensors has become a prominent research field.

HAR is commonly done by gathering signals from smartphones sensors and processing them through Artificial Intelligence (AI) algorithms for classification. However, during the process of data collection, the original data of sensors usually contains noise (missed value, error value or abnormal value, etc.) introduced by the interference of external environment^2^. The cumulative effects in long-time dependent sequence may lead to wrong classification. To avoid the problem, the existing AI models usually take manual interpolation or feature selection methods for the original data. However, these models are time-consuming and effort-consuming, and they depend on the experience of researchers more or less. Therefore, it is worth studying to design an automatic model to eliminate the influence of noisy data. Wang et al.^3^ investigated the state-of-the-art literature about sensor-based activity recognition based on machine learning. The results verified that the models based on CNN did well in features extraction. Thus, a serial of CNN-based models was worked out. Ronao et al.^4^ proposed a deep CNN model to perform efficient and effective HAR based on data of smartphone sensors. The model took use of the inherent characteristics of activities and extracted automatically features from 1D time-series signals. Ignatov^5^ proposed a novel CNN architecture, which could accept both the dynamic features of sensor data and the statistical features of HAR. The experiments showed that the proposed model had better performance than the baseline models. Andrade-Ambriz et al.^6^ designed a temporal CNN network for recognizing HAR that leveraged both spatial location features and temporal features. The results told that the accuracy of HAR recognition was improved. Gianni et al.^7^ proposed to generate HAR-Images from the raw data of accelerometer sensors and fed HAR-Images as fingerprints into CNN-based model for further handling. Gholamrezaii et al.^8^ proposed a new architecture that consisted solely of convolutional layers, which were removed the pooling layers and added strides instead. The computational time of model would decrease notably. Khan et al.^9^ proposed an attention-based multi-head model for HAR, which composed of three lightweight convolutional heads. The methodology leveraged 1-D CNN in the convolutional head to get features from sensors. Mahmud et al.^10^ proposed an architecture including multiple CNN feature extractors for HAR. Extracted features were optimized through a combined training stage or multiple sequential training stages. Ali et al.^11^ proposed the use of a combined CNN and Naive Bayes for accuracy and robustness to distinguish between false and true alarms of HAR. Lai et al.^12^ proposed 1-D dense attention neural network model for HAR. The model had the ability of frequency attention after applying attention mechanism.

The above models had good performance when extracting sptial location features. But they came across the problem of extracting temporal features. Thus, Amer et al.^13^ proposed two-step approach to recognize HAR. One was to transform the data into time-frequency representation by applying spectral analysis. The other was to classify the time-frequency representation using CNNs. With the emergence of LSTM network, it is playing an important role in recognizing long-time dependent sequence samples. Ai et al.^14^ investigated these existing classifiers for HAR. The results shown that LSTM-based classifiers had advantages on inferring the long-term human activities. Zhu et al.^15^ used LSTM network to learn long-term temporal representations from the trajectories of human skeleton joints. And they designed a fully connected deep LSTM network for end-to-end HAR recognition. Chen et al.^16^ conveyed complementary information by feature embedding method and fed those embeded features into a deep LSTM network to improve recognition accuracy. CNN-based network and LSTM-based network have their own advantages. Researchers integrated them for higher accuracy. Khan et al.^17^ proposed an ensemble model of CNN and LSTM. The model utilized CNN for spatial features extraction and LSTM network for temporal features extraction. Shalaby et al.^18^ presented deep learning model composed of CNNs, GRUs and LSTMs, which was used to extract high dimensionality and time sequence features. Shakerian et al.^19^ proposed a fuzzy *softmax* classifier based on CNN and LSTM. The classifier used CNN for extracting the high-level features of the sensors data, and then used LSTM for learning the time-series behaviors of the abstracted data. Yadav et al.^20^ proposed a novel deep convolutional long short-term memory model for HAR. The proposed model was a sequential fusion of CNNs, LSTMs, and fully connected layers. Thakur et al.^21^ proposed a DL-based unified model composed of CNNs, autoencoders, and LSTMs. The model learnt both spatial features and temporal features from smartphone sensor data. Gao et al.^22^ presented a new dual attention method, which blended channel and temporal attention on residual networks to improve feature representation ability of CNNs and LSTMs.

During the processing of HAR, time consumption is as important as accuracy. Nevertheless, according to^23^, these LSTM-based models spent a lot of time to handle long time-series problem. And they split the continuous time sensor data into samples for processing one by one. This kind of sequenced structure with corresponding splitting technology is obviously slower than parallel ones. At the same time, the accuracies of the mentioned models were low when facing noisy data. Thus, in this paper, we propose a novel CNN-based Bi-LSTM parallel model with attention mechanism for human activity recognition with noisy data. The contributions of this paper are as follows:

- We adopt a well-designed equal time step sliding window method for maximizing data utilization, splitting raw multi-variate data into serveral single-variate ones, and speeding up the frequency of detection.
- We propose a ConvBLSTM-PMwA model, which uses CNN-based bi-LSTM network for dimensionality reduction and elimination of noisy data; which uses a parallel structure for time complexity reduction; which also uses attention mechanism for high accuracy by redistribution of the weights of key representations.
- We compare the experimental results of ConvBLSTM-PMwA model with state-of-the-art models on two different HAR datasets. The ConvBLSTM-PMwA model has enhancement in both classification accuracy and computational time complexity, even if facing HAR data with noise.

The rest of the paper is organized as follows: In “Methods” section, the equal time step sliding window method and the architecture of ConvBLSTM-PMwA model have been expressed in detail. In“Results” section, datasets and the experiment environment are described, and the experiment results are shown in terms of the recognition accuracy and the time complexity. In “Discussion” section, the experimental analysis for the results is presented. Finally, in “Conclusions” section, the overall model is concluded with suggestive future enhancements briefly.

## Methods

**Figure 1:**
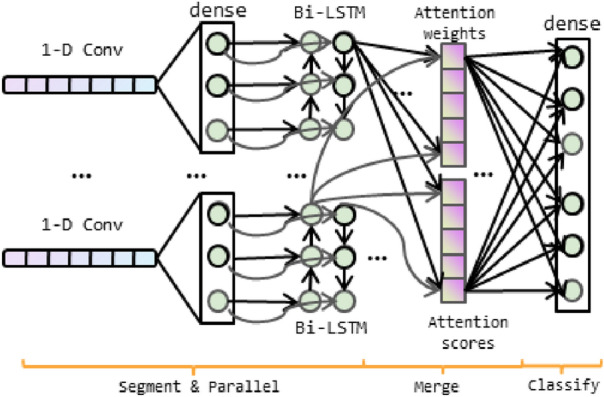
The block diagram of the proposed methodology.

In this section, a CNN-based bi-directional LSTM parallel model with attention mechanism is proposed and discussed including the tuning of training parameters detailed. As shown in Fig. 1, the original HAR data is segmented and fed into parallel 1-D CNN networks with $1 \times 3$ kernels for features extraction. The number of CNN-based bi-LSTM blocks *n* depends on the channels of HAR dataset. And then, the full connection layer activated by sigmoid is used for the purpose of feature representation. Next, the bi-LSTM networks are used to learn the valid information from noisy data in their hidden states through forward and backward propagation. Sequentially, an attention mechanism is used to merge important features together and choose the critical features by redistributing the weights. At last, a full connected layer is used for classification. Some key technologies are used in the ConvBLSTM-PMwA model to avoid over-fitting problem, such as the dropout layer and L2-normalization loss function.

### Segment and feature extraction

CNNs are used as dominant networks for the representation of HAR features. HAR data is captured as a sequential time series, and has *n* channels, such as body_acc-[x,y,z], body_gyro_[x,y,z] and total_acc_[x,y,z] etc. Every time step is related with the front and back ones. That is, HAR dataset is a time related information set, which can be represented by a matrix comprised by smartphone different sensor data in same time step. The shape and content of represent matrix looks like Eq. (1), where *A*, *G*, *M* are various sensors, *x*, *y*, *z* mean the directions of the sensors, and *t* represents the time step of activities.

1 $$\begin{aligned} \begin{bmatrix} A_{x0} &{} A_{y0} &{} A_{z0} &{} G_{x0} &{} G_{y0} &{} G_{z0} &{} M_{x0} &{} M_{y0} &{} M_{z0} &{} ...\\ A_{x1} &{} A_{y1} &{} A_{z1} &{} G_{x1} &{} G_{y1} &{} G_{z1} &{} M_{x1} &{} M_{y1} &{} M_{z1} &{} ...\\ ... &{} ... &{} ... &{} ... &{} ... &{} ... &{} ... &{} ... &{} ... &{} ...\\ A_{xt} &{} A_{yt} &{} A_{zt} &{} G_{xt} &{} G_{yt} &{} G_{zt} &{} M_{xt} &{} M_{yt} &{} M_{zt} &{} ... \end{bmatrix}. \end{aligned}$$

**Figure 2:**
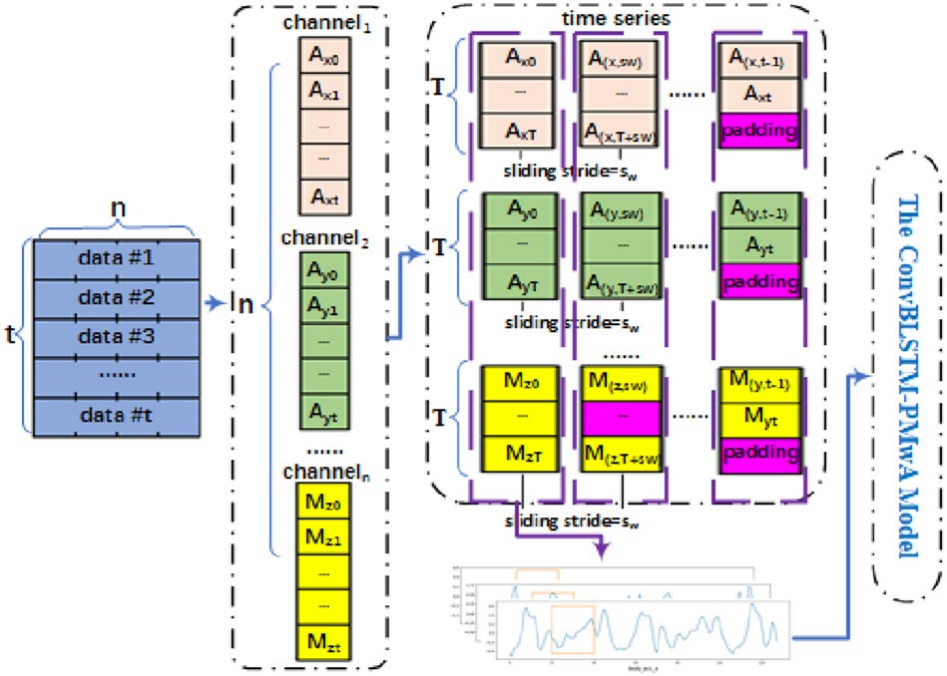
Equal time step sliding window method.

In order to speed up the classifying time, a parallel structure is worked out to deal with these long time-series signals. Corresponding to this, training data should be segmented reasonably. Thus, an equal time step sliding window method is proposed. As Fig. 2 shown, the shape of above matrix is *t* $\times$ *n*. Normally, the number of parallel units equals to *n*. That is, the partitions should be *n*. The time step sliding window is used to segment time series data into fixed-length sub-segments, and pad time series missed and agnostic data. Assume that the time step sliding window is *T*. Then the input shape of every CNN-based bi-LSTM blocks should be equal to (1, *T*). The sliding stride $s_w$ is used to locate the next position of detection sub-segments. If the first sub-segment starts from $t_0$, the next position would be $t_0+s_w$. The benefit in detection phase is that the sub-segments in given $s_w \times t_s$ can be sent for detecting in time without waiting for all the data of their whole lifecycle, where $t_s$ means sampled interval. And the benefit in training phase is maximizing data utilization. After above pre-processing, the original big matrix is translated to several smaller matrixes.

### Bi-directional LSTM

**Figure 3:**
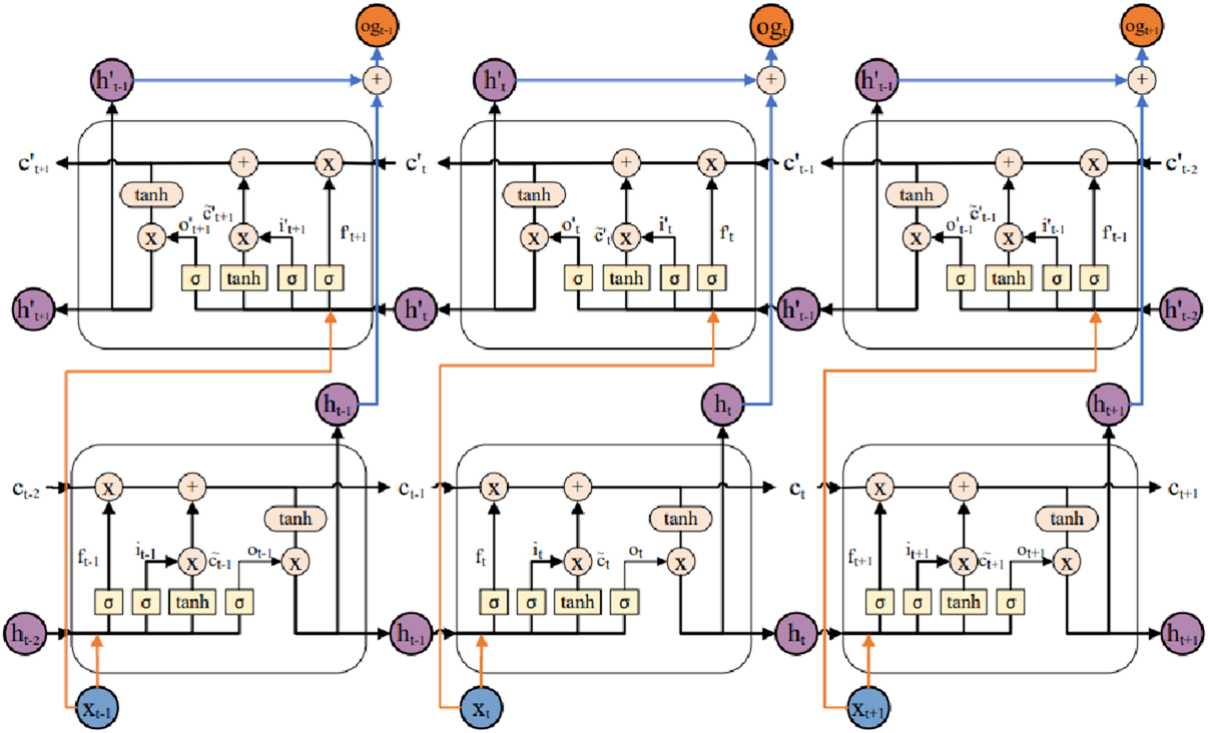
Basic structure of three-stage Bi-LSTM network.

The HAR data is a sequence of sensors signals with noise. Noisy data is hurt to the final predicting result. In order to estimate and eliminate these noise, Bi-LSTM network is adopted. Normally, LSTM network is for one-way time series data prediction. But Bi-LSTM network has two-way stacked LSTM network. As illustrated in Fig. 3, the forward LSTM network is for learning from the previous values in the forward direction, and the backward LSTM network is for learning from the upcoming values in the inverse direction. Both the learnt information by above hidden states is combined in latter layer. Equation (2) explains the operations performed in bi-LSTM unit,

2 $$\begin{aligned} \begin{aligned} h_t=\sigma (w_1x_t+w_2h_{t-1})\times tanh(C_t),\\ h'_t=\sigma (w_3x_t+w_4h'_{t-1})\times tanh(C'_t),\\ og_t=w_5h_t+w_6h'_t. \end{aligned} \end{aligned}$$

where $x_t$ is the input at time *t*. *w*’s are the weights of gates of LSTM cells. $h_t$ and $h'_t$ are the forward and backward output, respectively. The output gate $og_t$ keeps information about the bi-directional steps.

In this paper, the generated vector of chosen features by CNN and dense is fed to the bi-LSTM in parallel. A regularizer of L2 and an activation tanh are adopted to finish normalize, which can help to reduce the over-fitting. When running the network, the vector with a size of $n \times o$ are combined by the next layer, where *o* is the number of the hidden neurons of each bi-LSTM unit.

### Attention mechanism

**Figure 4:**
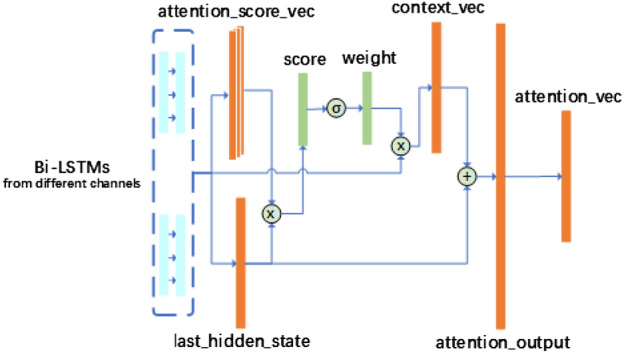
Attention mechanism of the ConvBLSTM-PMwA model.

In the attention layer, there are two tasks. One is to merge the output of the upstream layer. This task is simple. The layer needs only to put the output together in channel order, since the original input data to parallel structure contains the channel dependency. As known, the different representations are contributed differently. Thus, the other task is to filter the important representations out for the purpose of recognition. An attention mechanism^24^ is used to redistribute the weights of representations. As Figure 4 shown, firstly, attention mechanism calculates the last hidden state and attention score vectors based on inputted data from different channels of Bi-LSTM networks. Next, the scores of representations are gotten by dot-product function. Here, we adopt the dot-product attention, which benifits to time complexity. After this, a *softmax* function runs on these scores for getting the normalized ones. Next, they are aligned and summed up for context vectors by the Eq. (3),

3 $$\begin{aligned} \begin{aligned} \mu _t=tanh(w_d \times flatten(og_t's) + b_d),\\ \alpha _t=\frac{exp(\mu _t\mu _d)}{\sum _{t}exp(\mu _t\mu _d)}, \end{aligned} \end{aligned}$$

where $\mu _t$ means the representation information of hidden layer, $\mu _d$ is the similarity of feature vectors. $\alpha _t$ is the normalized weight.

### Other common layers in the ConvBLSTM-PMwA model

1-D CNN is being used since it can capture all the tiny changes in each time step. The changes are critical for sequenced learning in bi-LSTM for activity recognition. The layer is equipped with 64 filters and activated by *ReLU* nonlinear activation function. There is a full connection layer in the beginning and end part of model. They are playing different roles. The former is used to generate an input vector, and the latter is used to reduce the dimension of the features for producing the classification outcome. Thus, *sigmoid* and *softmax* activations are used for different purposes. To avoid the overfitting problem, a dropout layer with 0.6 dropout ratio is followed by the first dense layer, which permits the hidden layer to drop out certain neurons during training randomly. During model compiling, $categorical\_crossentropy$ loss function is used to calculate cost in training set and validation set. And *rmsprop* optimizer is used to adjust weights and biases through back propagation.

### Ethics approval and consent to participate

The data of experiments about human activities comes from the UCI and WISDM public datasets. The UCI HAR dataset is approved by UC Irvine, and the WISDM HAR dataset is approved by the Wireless Sensor and Data Mining lab. The authors confirm that all researches are performed in accordance with relevant guidelines and regulations.

## Results

In this section, we give the detailed description about the public UCI and WISDM datasets firstly. Next, we revise UCI dataset to the balanced one with noisy data, and keep WISDM as the unbalanced one without noisy data. And then, hyperparameters are well-tuned through testing the output of different values. At last, comparable experiments are carried out in terms of accuracy and computational time complexity by using the above two datasets.

### HAR datasets introduction and revision

To prove the effectiveness of the proposed ConvBLSTM-PMwA model, two public smartphone-based HAR datasets are adopted. One is the blanced dataset, and the other is the unblanced dataset.

**Figure 5:**
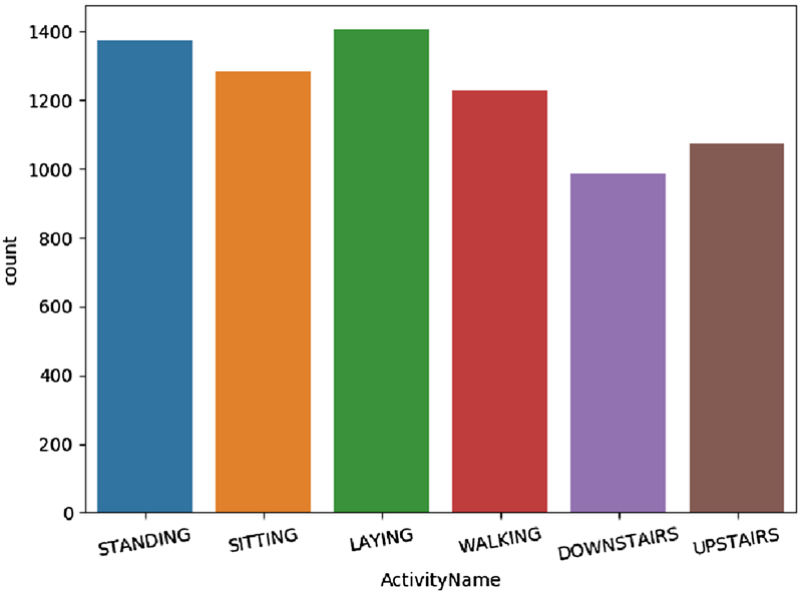
The distribution of activities on UCI HAR dataset.

**Figure 6:**
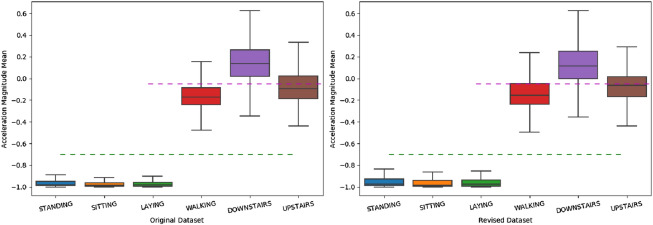
The boxplot of acceleration magnitude on original and revised UCI HAR dataset.

The UCI HAR^25^ is a public dataset, which is carried out with 30 subjects within an age between 19 and 48 years old. Each volunteer finished six activities wearing a smartphone (Samsung Galaxy S II) on the waist: walking, going upstairs, going downstairs, sitting, standing and lying. The data of embedded accelerometer and gyroscope sensors is sampled by the rate of 50 *Hz*. And then, the data is pre-processed and labeled manually. The size of samples is 128 with 50% overlap. At last, the training dataset is randomly partitioned into two small sets by 80%:20%. The 80% one is for training and the 20% one is for validation. In order to verify the ConvBLSTM-PMwA model, 10% values on both training and testing datasets are changed to 0 randomly, which is simulating the noisy data. And then, we process the check about data balance and exploratory data analysis. In Fig. 5, the numbers of different activities are between 1,000 and 1,400, the average number is 1,225, thus, the dataset is good for training and testing. It is not necessary to do undersampling or oversampling. In Fig. 6, the boxplot comparison of the original and revised data of the mean value of acceleration magnitude, as an example, is drawn. The minimum, maximum and Q2 are changed. But the activities can still be classified.

**Figure 7:**
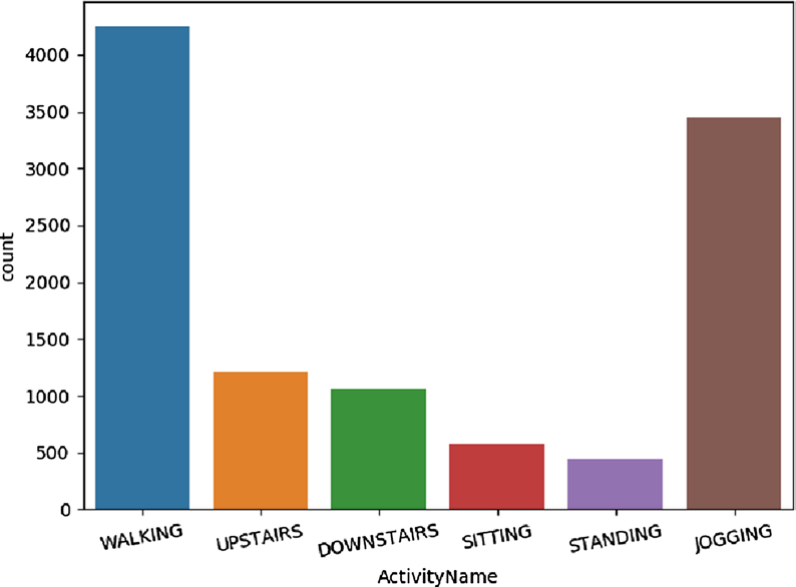
The distribution of activities on WISDM HAR dataset.

The WISDM HAR dataset^26^ is a standard public dataset provided by the Wireless Sensor and Data Mining lab. The dataset was collected from 36 subjects using smartphone accelerometer sensors. Each volunteer finished six activities, such as sitting, standing, walking, jogging, walking upstairs, and walking downstairs. The data of 3-axial linear acceleration measurements is sampled by the rate of 20 *Hz*. We use raw time series data, and split them into sub-segments every 128 samples. We split this dataset into training sets including the data from users 1-26 and test sets with the data from the rest 10 users. And the training sets is randomly partitioned into two small sets by 80%:20%. The 80% one is for training and the 20% one is for validation. As Fig. 7 shown, this is an unblanced dataset.

### Experiment environment setup

The experiment is finished on a laptop with Intel(R) Core(TM) i5-7200U CPU. GPU is not used here, since most smartphone has not high-performance GPU, too. But for the purpose of the comparison of time complexity, the same experiment environment is used, which is a machine with Intel Xeon E5-2695 v4 CPUs and NVIDIA Tesla M40 GPUs. The library keras with tensorflow as backend is imported into the ConvBLSTM-PMwA model implementation.

**Figure 8:**
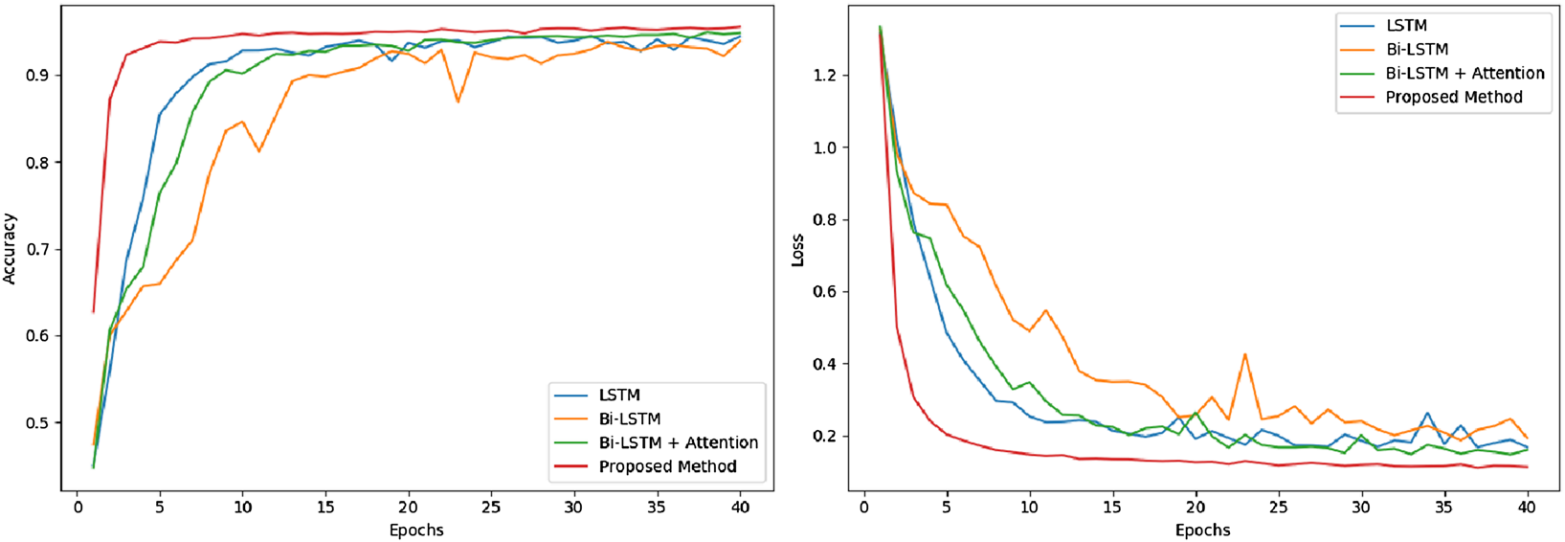
The accuracy and loss comparation of different models on validation set.

**Figure 9:**
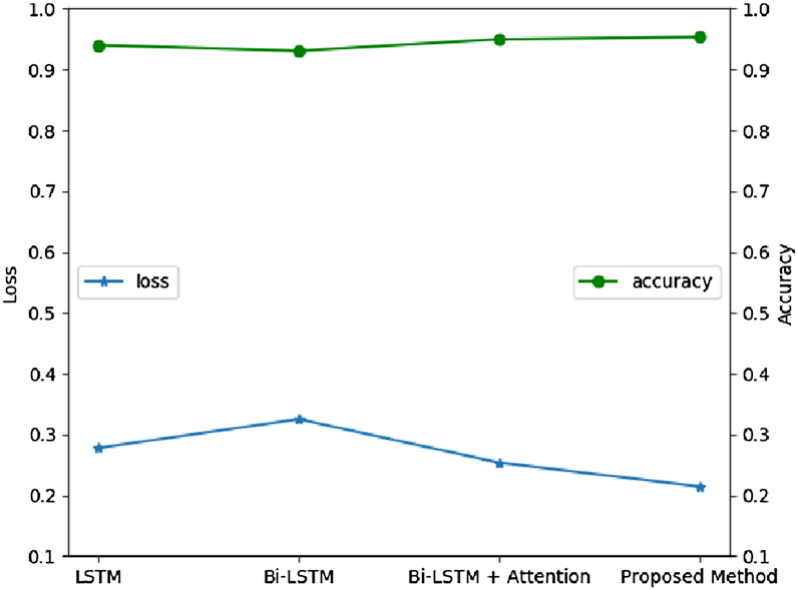
The accuracy and loss comparation of different models on test set.

The validation of bi-LSTM layer and attention mechanism is tested and compared with other RNN models on UCI HAR dataset. In Fig. 8, the accuracy and loss of LSTM, bi-LSTM, CNN-based bi-LSTM and CNN-based bi-LSTM with attention are compared. The training is done in 40 epochs. Obviously, the proposed CNN-based bi-LSTM with attention network has best performance, whose accuracy is as high as 95.6% and cross entropy loss is lower than 0.11. The epoch with best performance is chosen to validate on testing set. In Fig. 9, the results on testing set are listed. The ConvBLSTM-PMwA model still has best performance thinking about accuracy and loss at the same time. Its accuracy is 96.71%, which is little higher than CNN-based bi-LSTM models, but the loss is far better than them. Thus, CNN and attention are used and added into our proposed model.

**Figure 10:**
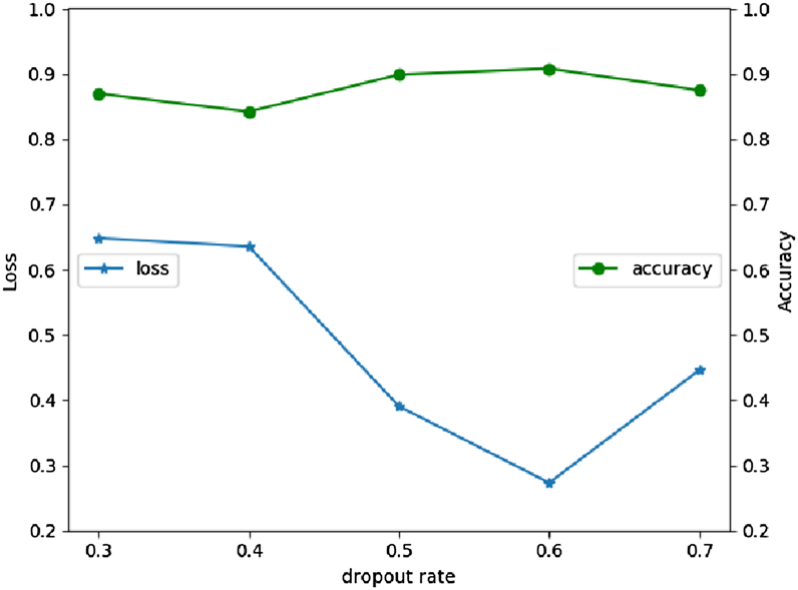
The accuracy and loss comparation with different dropout ratio.

**Figure 11:**
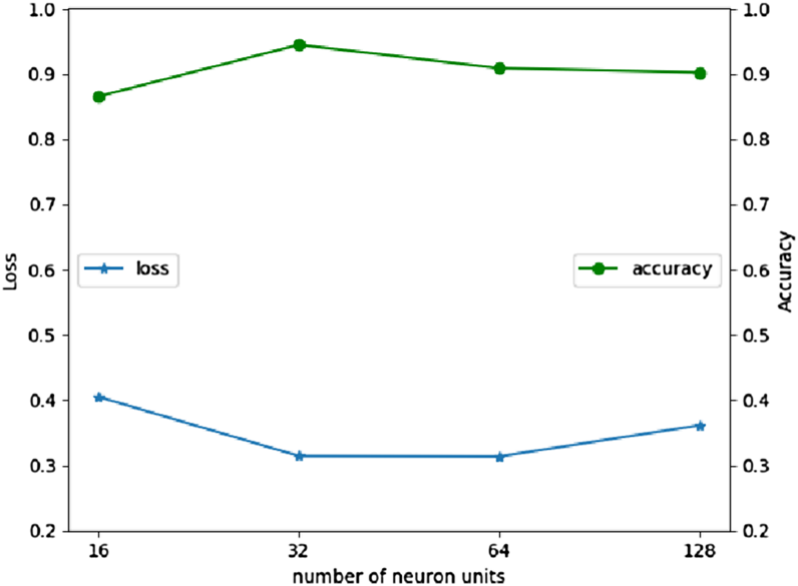
The accuracy and loss comparation with different neuron units.

After getting the model, hyperparameters are well-tuned through testing the output of different values. A greedy-wise tuning method named GridSearch is used to achieve the optimal performance as much as possible. The different dropout values and the number of neuron units are tested in UCI HAR dataset. As shown in Fig. 10, when dropout rate is 0.6, both the loss and accuracy have the best performance. Thus, we use 0.6 as the dropout rate in the ConvBLSTM-PMwA model. After applied the chosen dropout rate, different number of neuron units in 1D CNN layer is tested by GridSearch. As Fig. 11, the best number is 32. It can learn the representation features and get an accuracy of 94.46%. Learning rate is not necessary, since rmsprop is used. It tries to use the best learning rate automatically. The accuracy is increasing step by step during tuning process. Once the above exploration is done, the hyperparameters are gotten. Table 1 lists parts of the hyperparameters.

**Table 1:** Table of hyperparameters.

Hyper-parameters	Values
Number of parallel CNN-based Bi-LSTM units	80% of CPU cores
Hidden neurons of 1D CNN units	32
Batch size	16
Dropout ratio	0.6
Dominant layers	Bi-LSTM, Attention, Dropout

### Classification accuracy

**Figure 12:**
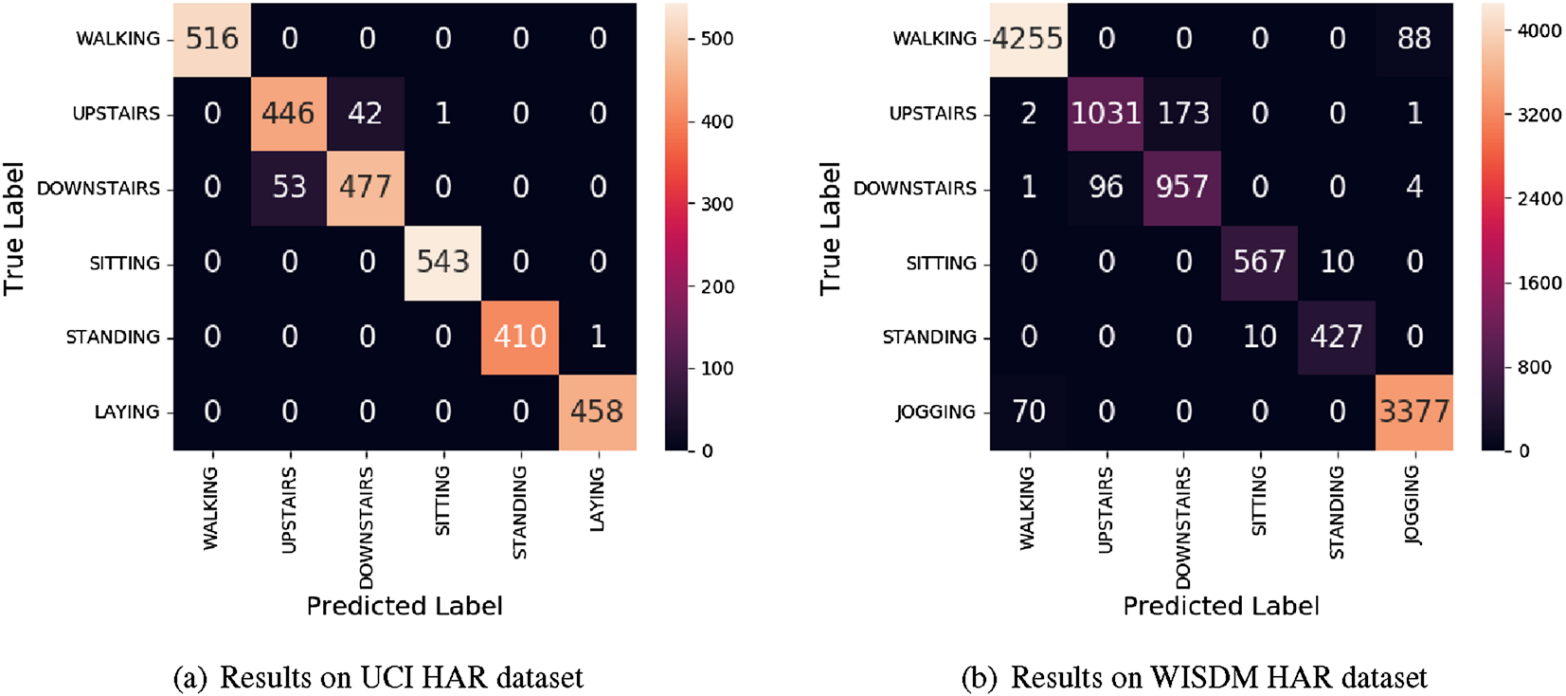
The heatmap of classification of the ConvBLSTM-PMwA model.

As Fig. 12 shown, they are the heatmaps of classification in revised UCI HAR dataset and WISDM HAR dataset. In Fig. 12a, The model on UCI HAR dataset has a good performance in walking, sitting, standing and laying. The predication accuracy can reach to 99.89%. As to upstairs and downstairs, there is a higher error rate. But the overall accuracy of 96.71% is good to do HAR. In Fig. 12b, The model on WISDM HAR dataset almost has the same performance, and get an accuracy of 95.86%. This shows that the proposed model is stable in both balanced and unbalanced datasets.

The comparison with other classic models is listed in Table 2. Obviously, the accuracy of the ConvBLSTM-PMwA model is a little better than these state-of-the-art models. The reason is that more feature extraction processes are executed by numerous transformations for obtaining variegated representations of the features encoded in raw data before feeding to the ConvBLSTM-PMwA model.

**Table 2:** The classification comparation on the UCI and WISDM datasets.

Models	Accuracy (%)	Accuracy (%)
	on UCI	on WISDM
Inherent Features-based CNN^4^	91.75	91.50
Coherent Features-based CNN^5^	92.31	92.20
HAR-image CNN^7^	93.27	91.64
Multi-stage CNN^10^	94.29	93.19
Attention-based Multi-head CNN^9^	95.62	94.90
Temporal CNN^6^	96.31	95.71
Feature Embedding-based LSTM^16^	96.31	95.55
ConvAE-LSTM^21^	96.41	95.60
Attention-based LSTM^22^	95.18	93.74
Hybrid CNN and LSTM^17^	96.55	95.65
Proposed Method	96.71	95.86

### Computational time complexity

**Figure 13:**
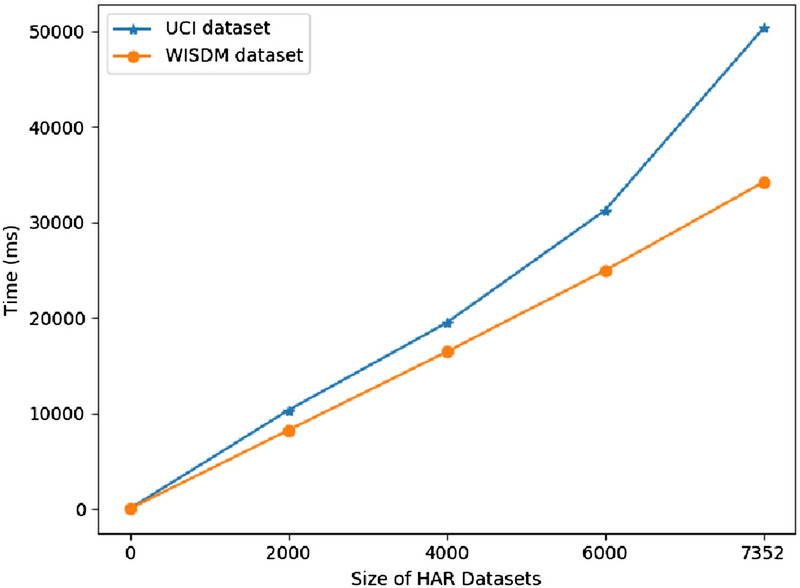
The time consumption on different scale HAR datasets.

The time consumption experiments are run in the mentioned laptop. As Fig. 13 shown, the size of dataset is divided by 2,000. The time consumption in above dataset size is experienced. From the results, with the increase of data size, the computational complexity is increased linearly. Thus, the ConvBLSTM-PMwA model is good on long time-series datasets.

The comparison of time complexity between different models on testing datasets is listed in Table 3. We shrink the scale of WISDM dataset, which has the same size with UCI dataset. Compared with deep CNN models, the ConvBLSTM-PMwA model performs better. Since we can see that the mean number of parameters in CNN-based bi-LSTM units are about 6,150, which is much smaller than deep CNN models. But compared with other parallel models, its time consumption is little higher, since the CNN network is introduced for feature extraction on a long time-series sample input.

**Table 3:** The comparison of time consumption among models on the UCI and WISDM datasets.

Models	Time (ms)	Time (ms)
	on UCI	on WISDM
Inherent Features-based CNN^4^	23.61	23.01
Coherent Features-based CNN^5^	25.61	24.22
HAR-image CNN^7^	27.46	25.31
Multi-stage CNN^10^	54.29	49.89
Attention-based multi-head CNN^9^	25.62	23.22
Temporal CNN^6^	16.31	13.57
Feature Embedding-based LSTM^16^	65.02	60.04
ConvAE-LSTM^21^	67.08	61.17
Attention-based LSTM^22^	75.18	72.61
Hybrid CNN and LSTM^17^	60.03	54.14
Proposed Method	14.71	12.11

## Discussion

In this study, we propose a novel CNN-based bi-LSTM parallel model with attention mechanism for human activity recognition with noisy data. During pre-processing, a well-designed equal time step sliding window method is used to split raw multi-variate data into several single-variate ones for parallel handling. And the method is also used to segment data into fixed sub-segments in time domain. Once the required data length of sub-segment is met, the HAR starts. This is maximizing data utilization on training phase, and speeding up the frequency of detection in real scenarios. CNNs are efficient in extracting representations from those single-variate data. And the output of CNNs is fed into bi-LSTM for getting temporal features. Considering these noisy data, bi-LSTM network is more accurate and effective. Since sensor data streams are split into different groups of channels, the representations from different CNN-based bi-LSTM network have different impact on final results. Hence, an attention mechanism is adopted for weighing these representations and assembling them together.

In computational time complexity, a parallel handling model is adopted. Time consumption of bi-LSTM network is grown quickly with the scale of inputted representations increasing. Hence, we split raw multi-variate data into several single-variate ones for dimensionality reduction firstly, and then, we use CNNs for automatic dimensionality reduction further. By reducing the numbers of inputted representations of bi-LSTM network, the ConvBLSTM-PMwA model gets recognition results in an acceptable amount of computational time.

Furthermore, we design classification accuracy and computational time consumption experimentations. These experimentations are done on two standard smartphone-based HAR datasets. To prove the effectiveness of the proposed ConvBLSTM-PMwA model, we compare our results with state-of-the-art models. The results show that the ConvBLSTM-PMwA model performs better than the existing CNN and RNN models in both classification accuracy (96.71%) and computational time complexity (1.1 times faster at least), even if facing HAR data with noise.

## Conclusions

HAR based on smartphones has a world-wide need, especially in human-computer interaction, covid-19 tracking and public safety etc. An equal time step sliding window method and a ConvBLSTM-PMwA model are proposed for recognizing human activity, even if facing HAR data with noise. Before experimentation, we tune the key hyperparameters in model by GridSearch method, and train the model with well-tuned hyperparameters. The proposed model can have a good performance in both accuracy (96.71% on the UCI dataset and 95.86% on the WISDM dataset) and time consumption (14.71 ms on the UCI dataset and 12.11ms on the WISDM dataset). But recently, we also notice that some small-scale AI networks for smart phone and other IoT equipment have come into the world. Next, we will try and integrate these ideas of classic mobile AI networks into our proposal. Maybe this will improve the time performance further.

## Data Availability

The datasets analysed during the current study are available in the UCI Machine Learning Repository, http://archive.ics.uci.edu/ml/datasets/Human+Activity+Recognition+Using+Smartphones, and in the WISDM Lab webpage, https://www.cis.fordham.edu/wisdm/dataset.php.
